# Supplementary material for: Identification of photoperiod-induced specific miRNAs in the adrenal glands of Sunite sheep (Ovis aries)
Source: Front Vet Sci. 2022 Jul 22;9:888207. doi: 10.3389/fvets.2022.888207 (PMC9354845; doi:10.3389/fvets.2022.888207)
Supplement: Supplementary file 1 [file Data_Sheet_1.ZIP › Supplementary file/miRDeep2 file.zip!/miRDeep2 file/Novel_Structure/LP42A2/Novel_354.pdf]

Provisional ID	:	NC_019458.2_151
Score total	:	0.8
Score for star read(s)	:	-1.3
Score for read counts	:	0
Score for mfe	:	0.5
Score for randfold	:	1.6
Score for cons. seed	:	
Total read count	:	19
Mature read count	:	19
Loop read count	:	0
Star read count	:	0

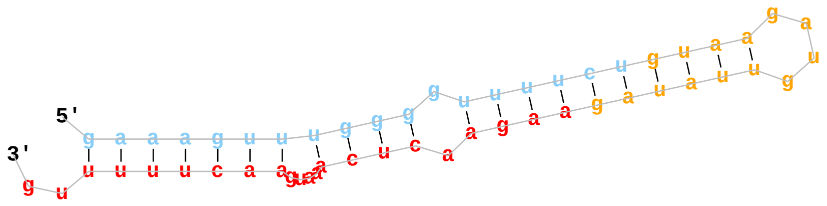

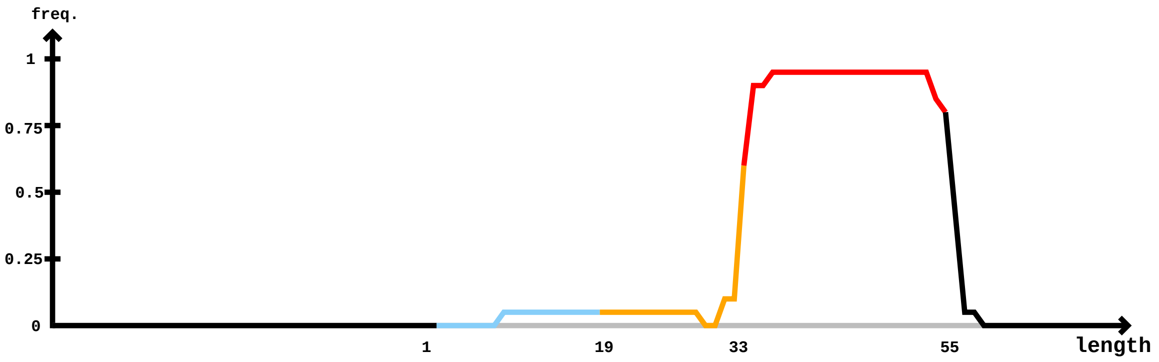

Star Mature

```
5'- aguguauccuuggacaucugcaaccuagaguagugccagaaaguuugggguuuucuguaagauguuauagaagaacucaaaugaacuuuuuggccagcccaauaccuuuc   -3'    exp
    ((.((((...(((....((((........)))).(((((((((((((((.((((((((((....)))))))))).))))....)))))))))))....)))))))))...    reads      mm         sample
    .............................................ugggguuuucuguaagauguu............................................    1          0          seq
    ....................................................................agaagaacCcaaaugaacuuuu....................    2          1          seq
    ......................................................................aaAaacucaaaugaacuuuuu...................    1          1          seq
    ......................................................................aaAaacucaaaugaacuuuuug..................    9          1          seq
    .......................................................................aAaacucaaaugaacuuuuug..................    6          1          seq
    .........................................................................aacucaaaugaacuuuuuggcU...............    1          1          seq
```
